# What is the perceived impact of Alexander technique lessons on health status, costs and pain management in the real life setting of an English hospital? The results of a mixed methods evaluation of an Alexander technique service for those with chronic back pain

**DOI:** 10.1186/s12913-015-0966-1

**Published:** 2015-07-28

**Authors:** Stuart McClean, Sam Brilleman, Lesley Wye

**Affiliations:** Department of Health and Applied Social Sciences, Faculty of Health and Applied Sciences, University of the West of England, Frenchay Campus, Coldharbour Lane, Bristol, BS16 1QY UK; School of Social and Community Medicine, University of Bristol, Canynge Hall, 39 Whatley Road, Bristol, BS8 2PS UK

**Keywords:** Pain management, Chronic back pain, Alexander technique, Complementary and alternative medicine, Service evaluation, Mixed methods

## Abstract

**Background:**

Randomised controlled trial evidence indicates that Alexander Technique is clinically and cost effective for chronic back pain. The aim of this mixed methods evaluation was to explore the role and perceived impact of Alexander Technique lessons in the naturalistic setting of an acute hospital Pain Management Clinic in England.

**Methods:**

To capture changes in health status and resource use amongst service users, 43 service users were administered three widely used questionnaires (Brief Pain Inventory, MYMOP and Client Service Resource Inventory) at three time points: baseline, six weeks and three months after baseline. We also carried out 27 telephone interviews with service users and seven face-to-face interviews with pain clinic staff and Alexander Technique teachers. Quantitative data were analysed using descriptive statistics and qualitative data were analysed thematically.

**Results:**

Those taking Alexander Technique lessons reported small improvements in health outcomes, and condition-related costs fell. However, due to the non-randomised, uncontrolled nature of the study design, changes cannot be attributed to the Alexander Technique lessons. Service users stated that their relationship to pain and pain management had changed, especially those who were more committed to practising the techniques regularly. These changes may explain the reported reduction in pain-related service use and the corresponding lower associated costs.

**Conclusions:**

Alexander Technique lessons may be used as another approach to pain management. The findings suggests that Alexander Technique lessons can help improve self-efficacy for those who are sufficiently motivated, which in turn may have an impact on service utilisation levels.

## Background

### Chronic back pain

Chronic back pain (upper and lower) is a very common disorder that affects around 1 in 3 adults in the UK each year [1, 2] and is frequently defined as pain that has persisted for more than 12 weeks (www.britishpainsociety.org) [3, 4]. Back pain is a significant public health issue [5], costing the UK NHS an estimated £480 million per annum. Research estimates costs to the UK economy due to back pain vary between £3 billion [6] and £12.3 billion per year [7], a variability that reflects the differences in definition of the disorder as well as what counts as costs. In the USA that figure is as high $100 billion in health care compensation and litigation [8], whilst in Australia a figure of $15 billion is quoted [9].

The process behind how general (and non-persistent) back pain becomes chronic is identified in the psychology of pain literature [10]. For most people (80-90 %) the pain diminish in a few days or weeks [11] but for some the pain becomes more long-term and distressing, and “multiple and overlapping problems” such as depression and anxiety are common [10]. Research highlights how disability and chronicity can develop through kinesophobia (fear avoidance of movement and pain), catastrophising and low self-efficacy of the individual [12]. However, it is difficult to ‘cure’ chronic back pain and conventional medicine has been ineffective in many ways [13, 14], contributing to a high level of patient dissatisfaction with medical care, hence treatments often aim at helping patients manage pain and reducing its effect on their lives [2]. Moreover, with no long-term effective medical treatment there is a considerable emphasis on self-management [3], including use of complementary and alternative medicine (CAM).

CAM use for chronic back pain is particularly prevalent in comparison to other long-term conditions [8, 15]; approximately 30 % of back pain sufferers use CAM [16, 17]. CAM encompasses a variety of treatments that exist outside of conventional (frequently biomedical) care, and for back pain most commonly used therapies include chiropractic, acupuncture, massage therapy, and yoga [18]. Alexander Technique (AT) is often perceived as a CAM modality, sitting as it does outside of conventional medicine [19].

### Alexander technique

Alexander Technique (AT) has been used extensively in diverse fields of performance such as acting and music [19], and is a primarily educational approach to managing posture and movement: “AT teachers combine hands-on guidance and verbal explanation to show individuals how to diminish self-damaging postural and movement habits, and to modify habitual responses to stimuli, which can include pain and stress.” [20]. Because AT is educational, practitioners are called ‘teachers’ and those who receive instruction are called ‘students’ (or pupils).

The Society of Teachers of Alexander Technique (STAT: www.stat.org.uk) recommends one-to-one Alexander lessons to discover if a ‘student’ has some postural issue or movement or other habit or misunderstanding that is causing or aggravating their problem, such as chronic pain. Opportunities are provided for self-observation and practice during closely monitored activities with constructive feedback provided by the teacher. Frequent hand contact from teachers is used to observe and interpret subtle changes in muscle tone and co-ordination and also to convey non-verbal information to the student. Students are encouraged to spend time each day (15-20 min) lying semi-supine while practising the Alexander mental ‘directions’, and incorporate what they have learned in their everyday activities [21].

### Evidence

An evidence base is emerging about the effectiveness of CAM for chronic back pain [9], but significant gaps exist. In 2007 a short article [17] reviewed the state of the art of CAM therapies for back pain and concluded that despite the weight of evidence increasing and the direction of travel of research being towards the positive, that more research into effectiveness was required, with AT mentioned as being particularly welcome due to its relatively low cost and associated low risk.

Previous studies have explored the effectiveness of AT lessons for chronic pain [22, 23]. A well-conducted factorial randomised controlled trial (ATEAM) suggested that AT lessons were clinically and cost effective for patients with chronic or recurrent back pain and could lead to a significant reduction in pain [23]. A comparison of six versus 24 AT lessons as adjuncts to massage therapy, and advice from a doctor to take exercise with nurse delivered counselling, found that six one-to-one AT lessons followed by an exercise prescription had long-term benefits, with a significant reduction in days in pain and disability. Although 24 one-to-one AT lessons led to better results, the economic analysis found that the best-value intervention was 6 lessons in AT with exercise prescription [24]. In addition, a systematic review of the evidence of effectiveness and safety of AT in health related conditions more generally [20] found that there is strong evidence for the effectiveness of AT lessons for chronic back pain.

### Aim of this evaluation

Given the robust controlled trial evidence about the clinical and cost effectiveness of AT lessons, the next step was to study AT in the ‘real world’. The aim of this mixed methods service evaluation therefore was to explore the role and perceived impact of AT lessons at a hospital out-patient Pain Management Clinic in the UK, including clinicians’ perspective and service users’ (n = 43) experiences of the lessons and their perceptions of benefits. For the purposes of this paper, where AT was delivered in a healthcare setting, we have chosen the term ‘service user’ as opposed to ‘student’.

## Methods

This study is a mixed methods evaluation with a before-and-after study design. The study took place at the Pain Clinic at St. Michael’s Hospital, University Hospitals Bristol NHS Trust from June 2010 to May 2011. The majority of the quantitative data collection preceded the qualitative. Qualitative research was undertaken to explore both service users’ and professionals’ experiences in-depth and help explain the quantitative results. Ethical approval for the study was obtained from the University of the West of England Faculty ethics committee and from the University Hospitals Bristol R&D Trust as NRES approval is not required for service evaluation studies.

### Referral route

At the time of the study, patients were referred to the Pain Clinic by their GP, where they saw one of four consultants who assessed the patient to see what treatment was suitable, such as medication, injections, psychological therapy, TENS, physiotherapy or acupuncture (as well as possible referral for a surgical opinion). The options also include a pain management programme for patients who have come to the end of the line in terms of treatment options. Using clear criteria for referral, the consultant referred patients with chronic or recurrent back pain who were not getting better, were not responding to conventional treatment and expressed an interest in AT lessons. All referred AT patients accepted an invitation to be part of the evaluation and were therefore included in the study.

Once referred, the service user received six one-to-one AT lessons with a qualified and experienced STAT registered AT teacher, over a period of six consecutive weeks. The lessons took place in one of the treatment rooms at the Pain Clinic. Each AT session lasted on average 40-45 min in duration, although the first lesson lasted longer as a first consultation. At the first AT lesson service users were asked by the AT teachers to sign a consent form agreeing to the evaluation study. Consent was re-established by an independent researcher at the last follow up time point.

### Quantitative data

Three questionnaires were administered at baseline, six weeks and three months. Service users completed the questionnaires at baseline and at 6 weeks in the pain clinic, just before starting their Alexander Technique lesson. SM, an independent researcher, administered the questionnaires at the 3 month time-point initially through the post. Postal non-respondents were followed up with telephone calls to administer the last set of questionnaire data. The data for all questionnaires were entered into an Excel spreadsheet by an administrator.

With regard to health status, the outcome tools were the Brief Pain Inventory (BPI) and the Measure Your Medical Outcome Profile (MYMOP). The Brief Pain Inventory was selected for assessing pain as it was the standard tool used in the hospital pain clinic and the data collected could be comparable to other pain clinic patients [25]. Primarily applied to assess cancer-related pain, the BPI has been tested for reliability and validity [25]. The BPI questionnaire consists of two parts: 1) pain severity, and 2) pain interference with seven domains of functioning (general activity, mood, walking ability, normal work, relationships, sleep and enjoyment of life) on a scale of 0-10. For patient identified health outcomes we used MYMOP, which has been tested for reliability [26]. This questionnaire was selected to investigate how well the lessons met service users’ objectives. Respondents write down two symptoms that bother them the most (symptom 1 and 2), an activity is limited by their primary symptom, and score how bad each symptom is on a 7-point Likert scale. (http://www.bristol.ac.uk/primaryhealthcare/resources/mymop/index.html). The same symptoms and activity are measured in follow up administrations of MYMOP.

To understand resource use, we selected the Client Service Resource Inventory. Widely used in economic evaluations, respondents record their NHS and personal costs. We calculated costs at a standard weekly rate (in £) and then compared across the three time points. The data for total costs included personal and NHS costs; the data for condition, non-condition and intervention costs were based on NHS costs only.

#### Data analysis

Two independent statisticians analysed the data: one analysed resource data from the Client Service Resource Inventory, and the other analysed the health outcome tools. Analysis was performed using Stata Version 11.2.^1^ For the health outcome tools, after checking for normality, the data for the BPI and MYMOP were analysed separately. For each dataset, we were interested in changes: between baseline and 6 weeks, baseline and three months, and between 6 weeks and three months.

For each outcome measure, we calculated mean values at each time point and also the mean change between each pair of time points (baseline and 6 weeks, baseline and 3 months, 6 weeks and 3 months). For all mean values we calculated the associated 95 % confidence interval (CI), by using the means standard error x 1.96. These confidence intervals were used to compare the differences of the means in the groups. Additional analyses were conducted with MYMOP. The symptoms and activities identified by patients were classified, grouped and totalled. Data on length of time with condition was grouped according to the MYMOP questionnaire categories: short term (less than one year), medium term (1-5 years) and long term (more than five years).

### Qualitative data

Qualitative data was collected from a sample of service users (n = 27) in telephone interviews, and from professionals (three clinicians and all four AT teachers) in face-to-face interviews.

SM interviewed service users at the three month time point, using a purposive sampling strategy of maximum variation. This included service users who completed the majority or all six lessons and fell into one of three groups: 1) showed little or no improvement in pain, 2) showed minimal improvement at six weeks or three months, and 3) showed great improvement at six weeks and at three months. The sampling strategy aimed at some demographic variation, taking into account age and gender, as well as length of time that they had experienced pain. The sample did not include those who were referred and did not attend (n = 1) or those who dropped out after one AT lesson (n = 1), as the numbers were insignificant. Service users were asked about the benefits and drawbacks of the lessons, referral route, experiences of the lessons and whether they had maintained the exercises after stopping lessons.

In gathering professionals’ views, SM conducted face-to-face interviews at the pain clinic with AT teachers on their views of the benefits and drawbacks of an AT service in an NHS pain clinic, and with clinicians (two consultants and a specialist nurse), about their views on the service, re-referrals to the pain management clinic, as well as potential improvements and recommendations.

Interviews lasted between twenty one and thirty eight minutes, and were audio-recorded with the permission of the interviewee.

#### Data analysis

**Fig. 1:**
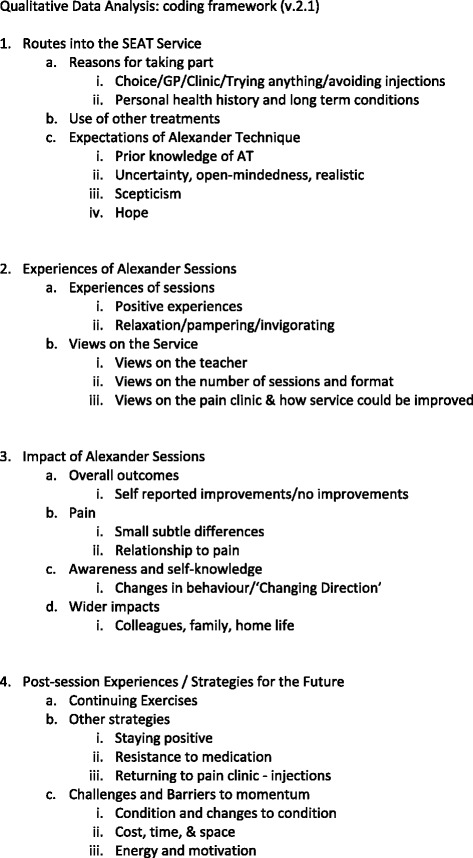
Coding Framework

Qualitative data was analysed using a thematic content analysis approach, coded to identify themes and categories, using constant comparison [27] to look for similarities and differences across the accounts to identify patterns and search for ‘deviant cases’ [28]. Initial open coding of individual transcripts generated a coding framework (see Fig. 1), which was amended as new data were gathered. Codes were gradually superseded by broader analytical categories, and through comparison across transcripts themes were identified. The analysis, data coding and organisation was aided by the software NVivo 9.

## Results

### Quantitative results

#### Characteristics of the sample

A total of 43 service users (female: n = 27) returned questionnaires at baseline, 41 (95 %) at 6 weeks and 39 (91 %) at 3 months. In total 41 (95 %) completed the AT lessons of whom 39 (91 %) completed the questionnaires at 3 months. The overall mean age was 53 (range 24 to 81; sd = 15.0086); data on age is not known for 12 respondents. This may be due to AT teachers administering the questionnaires at baseline and not insisting on completing some sensitive information such as date of birth.

#### Brief Pain inventory (BPI)

**Fig. 2:**
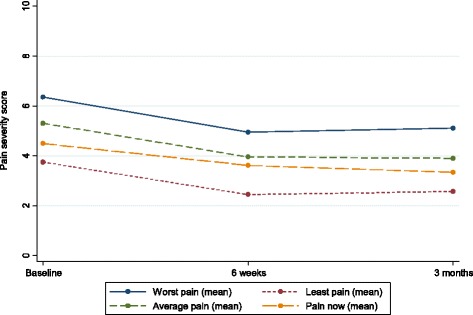
Brief Pain Inventory Severity Scores

**Fig. 3:**
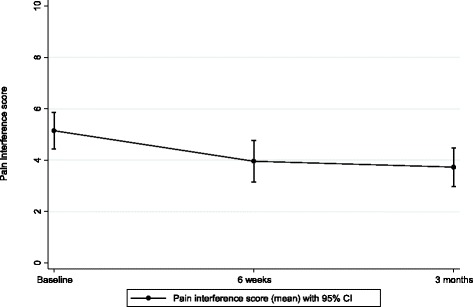
Brief Pain Inventory Interference scores

Results of the BPI showed a trend in the reported reduction of mean pain severity from 5.0 (ten-point scale) to 3.7 [-1.20 (95 % CI: -1.66 to -0.75)], and these scores were maintained at three months [-1.12 (95 % CI: -1.65 to -0.58)] (see Fig. 2 and Table 1). Similarly, BPI pain interference scores show a reduction in mean from 5.1 to 4.0 [-1.10 (95 % CI: -1.62 to -0.59)] on a ten-point scale at 6 weeks, and these scores were maintained at three months [-1.17 (95 % CI: -1.69 to -0.65)] (see Fig. 3 and Table 1). In summary, service users reported a modest reduction in pain severity and pain interference of roughly one point on a ten point scale.

**Table 1:** Brief pain inventory severity and interference scores^2^

	Baseline	6 weeks	3 months	Difference baseline to 6 weeks	Difference baseline to 3 months	Difference 6 weeks to 3 months
Number	43	41	39	41	39	38
Mean severity score (95 % CI)	5.0 (4.4 to 5.6)	3.7 (3.1 to 4.4)	3.7 (3.0 to 4.4)	−1.2 (-1.66 to -0.75)	−1.12 (-1.65 to – 0.58)	0.07 (-0.37 to 0.5)
Mean interference score (95 % CI)	5.1 (4.4 to 5.9)	4.0 (3.2 to 4.8)	3.7 (3.0 to 4.5)	−1.10 (-1.62 to -0.59)	−1.17 (-1.69 to -0.65)	0.02 (-0.34 to 0.39)

^2^Mean (paired) difference is not calculated on the same set of patients as the raw mean because not all participants have measurements at both time points (and therefore we don’t observe a value for the change for these participants). Note that there are 39 participants with data at both baseline and 3 months, which is fewer than the number of participants at either one of the individual time points, hence difference between two figures e.g. 5.1-3.7=1.4 but in table reported as 1.17

In comparing the separate domains from baseline to three months, the greatest reported shift was in mood, walking ability, normal work, sleep and enjoyment of life. Interestingly, between six weeks and three months, reportedly mood continued to improve with a decrease in mean score of 0.9 points (see Table 2).

**Table 2:** Brief pain inventory interference mean scores (with 95 % confidence interval) for each domain over time

	Baseline (95 % CI)	6 weeks (95 % CI)	3 months (95 % CI)
General activity	5.3 (4.9 to 5.8)	4.2 (3.7 to 4.7)	4.3 (3.8 to 4.8)
Mood	5.4 (4.9 to 5.9)	4.5 (4.0 to 5.0)	3.7 (3.2 to 4.2)
Walking ability	4.6 (4.0 to 5.1)	3.4 (2.9 to 3.9)	3.1 (2.6 to 3.6)
Normal work	5.9 (5.5 to 6.4)	4.1 (3.6 to 4.6)	4.2 (3.8 to 4.7)
Relationships	4.1 (3.6 to 4.6)	3.5 (3.0 to 4.0)	3.3 (2.8 to 3.7)
Sleep	5.1 (4.5 to 5.6)	3.8 (3.3 to 4.4)	3.6 (3.1 to 4.1)
Enjoyment of life	5.5 (5.1 to 6.0)	4.2 (3.7 to 4.6)	3.8 (3.4 to 4.3)

#### Measure yourself medical outcomes profile (MYMOP)

**Fig. 4:**
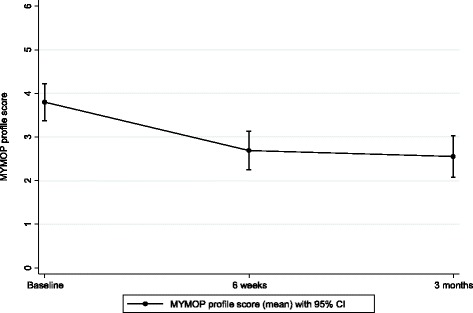
MYMOP Profile Score

The second health outcome questionnaire, MYMOP, showed that scores improved in terms of symptoms, activity and wellbeing by about one point on a seven-point scale, which was maintained at three months. The score for activity improved the most; this included movement, sports, gardening and sleeping. The overall MYMOP profile score improved on average by 1.14 units (95 % CI: 0.71 to 1.56) (see Fig. 4 for MYMOP profile score; Table 3 lists MYMOP scores across domains).

**Table 3:** MYMOP scores

	Baseline	6 weeks	3 months	Difference baseline to 6 weeks	Difference baseline to 3 months	Difference 6 weeks to 3 months
Number	43	41	39	41	39	38
Symptom 1 (95 % CI)	3.9 (3.4 to 4.3)	2.9 (2.4 to 3.4)	2.9 (2.3 to 3.6)	−0.85 (-1.27 to -0.44)	−0.87 (-1.49 to -0.26)	0.03 (-0.31 to 0.36)
Symptom 2 (95 % CI)	3.9 (3.5 to 4.4)	2.8 (2.3 to 3.4)	2.7 (2.1 to 3.3)	−1.06 (-1.47 to -0.65)	−1.06 (-1.55 to -0.58)	−0.03 (-0.42 to 0.35)
Activity (95 % CI)	4.2 (3.7 to 4.7)	2.7 (2.2 to 3.2)	2.6 (2.0 to 3.1)	−1.32 (-1.74 to -0.89)	−1.41 (-2.03 to -0.78)	−0.17 (-0.58 to 0.25)
Wellbeing (95 % CI)	3.4 (2.9 to 4.0)	2.4 (1.9 to 2.9)	2.0 (1.5 to 2.5)	−1.00 (-1.38 to -0.62)	−1.36 (-1.85 to -0.87)	−0.34 (-0.72 to 0.03)
MYMOP profile (95 % CI)	3.8 (3.4 to 4.2)	2.7 (2.3 to 3.1)	2.6 (2.1 to 3.0)	−1.02 (-1.32 to 0.72)	−1.14 (-1.56 to -0.71)	−0.09 (-0.35 to 0.16)

MYMOP also provided useful data on pain medication. A total of nine service users (21 %) did not take medication for pain throughout the time period of the evaluation (see Table 4). Of the 34 (79 %) who did take medication, 12 (28 %) reduced and 10 (23 %) stopped their medication between baseline and three months. Of the 34 service users (79 %) who were taking medication at baseline, two reported using alcohol for pain relief, one mentioned cannabis and one Chinese medical herbs. Prescribed pain medications included diclofenac, solpadol, cocodamol, tramadol, meloxicam, gabapentin, morphine and codeine and named over the counter medications were paracetamol, ibuprofen and aspirin. Nine service users (21 %) also mentioned prescription medications for mood and sleep such as amitriptyline (n = 6), diazepam (n = 2) and zopiclone (n = 1).

**Table 4:** MYMOP medication

Direction of change	Number of service users
Reduced	12 (28 %)
Stopped	10 (23 %)
No medication at baseline, 6 weeks or 3 months	9 (21 %)
No follow up data at 6 weeks or 3 months	8 (19 %)
No change	4 (9 %)
No medication at baseline & started at 6 weeks or 3 months	1 (2 %)
Variable	1 (2 %)
Not clear	1 (2 %)

#### Client service resource inventory

The Client Service Resource Inventory tracked costs. The wide confidence intervals suggest that there was substantial variation in this small sample.

**Fig. 5:**
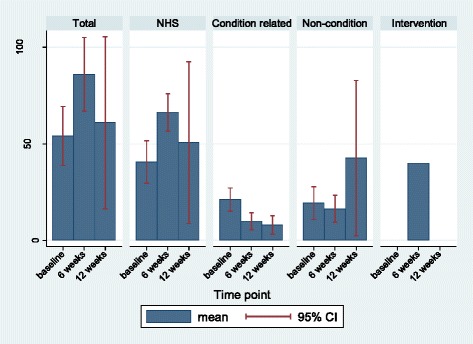
Total weekly mean costs

Total costs (NHS + personal costs) over the course of the study remained relatively unchanged with a baseline mean of £54.06 (95 % CI: £38.85 to £69.28) and a 3 month mean of £60.96 (95 % CI: £16.41 to £105.50) (see Fig. 5 and Table 5). At the six week time-point, total costs were unsurprisingly higher when AT lessons were included (6 week mean of £85.88 95 % CI: £66.78 to £104.99). Condition related costs for pain for these service users fell from a baseline mean of £21.16 (95 % CI: £15.21 to £27.11) to a three month mean of £7.98 (95 % CI: £3.16 to £12.79).

**Table 5:** Mean weekly costs (£) and associated 95 % confidence interval

	Baseline (n = 38)	6 weeks (n = 35)	3 months (n = 31)
Total costs	54.06 (38.85-69.28)	85.88 (66.78-104.99)	60.96 (16.41-105.50)
NHS costs	40.57 (29.65-51.49)	66.34 (56.65-76.02)	50.60 (8.76-92.44)
Condition costs	21.16 (15.21-27.11)	9.96 (5.59-14.33)	7.98 (3.16-12.79)
Non-condition	19.41 (10.91-27.91)	16.38 (9.29-23.47)	42.62 (2.44-82.81)
Intervention costs	0	40.00 (40.00-40.00)	0

### Qualitative results

Qualitative data were collected from service users and professionals including AT teachers and specialist pain clinicians. The following broad themes were elicited from the data: experiences of the lessons and the pain clinic (users and AT teachers only); impact of AT lessons (i.e. relationship to pain, comparison with other types of treatment for pain) (all participants) and future strategies, such as offering CAM to NHS service users (all).

Central to service users’ positive experiences of the lessons was the AT teacher. Service users said the teachers were supportive, knowledgeable, made a good connection with the service user, and had “healing hands”. Some service users focused on the ‘feel good’ factor,

*“I did feel incredibly mellow after the sessions… I felt like I was walking on air, it was brilliant. I felt about 2 inches taller and I just felt that everything was at peace with itself, there were no sort of aches and pains jangling for attention”*. (SEAT 121)

With regard to impact of the lessons, the majority of service users interviewed commented that the changes to health status were not necessarily very noticeable or large, that there was a subtle change to the pain; small differences had an impact on them.

*“I guess you never know completely… they are very subtle small changes that I’ve made but I mean I have not had another crisis point since then so it looks as if it would be down to that”*. (SEAT 101)

Pain levels had decreased or leveled off for most, but for some the pain had not changed significantly. But the AT lessons had an impact in how service users experienced pain. AT lessons enabled service users to manage the pain differently, and changed their perceptions of what the pain meant, as well as prevented pain from escalating:

*“It’s possibly helped me to breathe through the pain,…The Alexander Technique woman was telling me that I kind of close down with the pain, I just shut down to it, so I’ve learned to breathe through the pain. I’ve been doing the exercises she’s taught me to do but I wouldn’t say the pain has stopped”* (SEAT 144)

*“the big shift has been my relationship with the pain and acknowledging that when it’s there I have then got a choice to carry on having pain or I could just breath and relax and let it go and quite often that's the thing that makes the difference...... you know how am I sitting, how am I standing, and what am I doing? Because quite often I take on a lot and… it’s almost like a… it’s not constant meditation, I am not that good (laughs), but you know every now and then I check in and go oh ok slow down um and that's been just fantastically important to me”*. (SEAT 115)

Part of this can be attributed to patients reporting a wider range of coping strategies:

*“I am managing the pain much better now… with the tools given to me I feel more in control”* (SEAT 147)

Service users were asked how much they practiced AT and what strategies they used in order to improve. Those who viewed the Alexander Technique lessons as a “lifestyle change” with commitment to regular practice and learning more about the body, rather than a treatment option that was ‘done-to’ them, appeared to benefit substantially.

*Alexander Technique isn’t a quick fix; it’s a lifestyle change that you make…that’s how I view it anyway, to just help you along a little bit. I’ve been reading books and things....I don’t want it to go away, and I want to keep it there, so I know about it....I want to keep it fresh in my mind.* (SEAT 147)

The majority said that they tried to carry on what they had learned, though for some keeping up the momentum was difficult, as they needed a teacher to guide and motivate.

*“I try to do them in the evenings but without her (AT teacher) doing it with you its quite difficult”* (SEAT 124)

In the interviews with the clinicians once the quantitative results were known, they compared the results with other psychological therapies for chronic pain where there were similarly favourable results:

*“I’m pleasantly surprised about how good it is… [the service users] not massively better in terms of pain, but they seem to have enjoyed the experience, and they seem happier.... their wellbeing is improved despite the fact the pain isn’t much different…if more than half of them have significantly reduced their medication and they’re happier, and their pain is unchanged or slightly better, then that’s a very good result…to get either no change in pain or a slight improvement in pain on half as much drugs makes the difference in pain scores much more meaningful… If you look at psychological interventions for pain there is lots of evidence, and it’s almost always positive, but it doesn’t usually improve the actual pain scores very much. It will improve all the things like this has shown like quality of life and general wellbeing and health care utilisation and drug use, all those things are improved rather than the actual pain itself. Most people think if you go to a pain clinic, what do you want - you want your pain reduced, but in the majority of times that probably doesn’t happen, but if people’s function and feeling of wellbeing and quality of life and all those other things are improved then that means we’ve done a good job”*. (Consultant)

The clinicians mentioned that a few service users had requested re-referral to the pain clinic due to inability to cope with pain, yet even those that returned spoke favourably of the AT approach in terms of being a positive experience.

In the interviews with the AT teachers a key theme emerged about the importance of helping people with severe chronic pain, and teaching a service user that they would rarely come across in private practice:

*“…[it is] a big shift because you know I might have one person like that, you know one person with extreme chronic pain, maybe out of every 15 people I see… people with you know histories… I was teaching here somebody with 30 year history of pain from old injuries kind of constant chronic pain over decades I would not meet very often. So to actually work in a kind of setting where everybody presented with a very complex pain history… [when] you have that much pain… you become a different person, of course it shapes you. So I would say I have met very complex people in this setting at the pain clinic”*. (Teacher 2)

Also, the teachers spoke of the privilege of being able to work in the NHS context,

*“I would say it’s been a real privilege you know for me to work with people who perhaps couldn’t afford to have Alexander lessons, who wouldn’t afford to come and see me in the [private] clinic in Bristol, and feeling that in a way that's where I would really like to work with people who can’t access this kind of service”*. (Teacher 3)

## Discussion

A key finding of this study is that, while quantifiable changes in pain severity and interference scores were reported, more striking was how service users said they modified their ways of managing the sensations of pain, because of the AT lessons. As such, some service users reported no difference in levels of pain but did report reduction in pain medication and resource use, and consequentially in costs. According to at least one consultant, this signified ‘success’ as generally, service users do not eliminate their pain through the treatments offered by the pain clinic, but instead sufferers find alternative, more effective ways of self-managing symptoms.

### Comparison with original trial evidence

The findings of a major randomised controlled trial of Alexander Technique for chronic back pain found that 6 lessons plus exercise was almost as effective as 24 AT lessons and more effective than exercise alone [23]. Furthermore, the trial researchers noted that the benefits on chronic pain were still in effect up to 12 months after the lessons were delivered [23]. In our study in the naturalistic setting, where 6 lessons of AT were delivered *without* exercise, users reported a modest reduction in pain. However similarly, their scores were maintained after treatment delivery stopped. This suggests that although diluted within the naturalistic setting (possibly because of the lack of an exercise component), some chronic back pain sufferers may perceive that 6 AT lessons on their own offer useful benefits. This finding might support RCT findings that AT lessons without exercise are less effective, though some patients perceived improvement.

### Alexander technique and self-management of symptoms

Qualitative results suggest that service users were encouraged to be self-reliant and develop breathing and postural techniques for self-managing pain. Service users improved self-efficacy, becoming more effective over time at managing their pain. For example, AT teachers encouraged a more active ‘listening’ to the body and awareness of the embodied signs of pain and distress, and users developed techniques to manage that. This also led to some behavioural changes and changes in awareness and self-knowledge, with service users more actively involved in their health condition. Such findings contribute to a range of debates, not only about the value and impact of self-management techniques for pain [29], but also the mechanisms behind the effectiveness of self-management initiatives. Our study highlights the importance of self-management as a “dynamic process” [3] combining personal ‘subjective’ experience of pain with professional education (AT).

This study also shows that AT lessons may have similar impact to other educational and psychological interventions [30, 31], helping to reduce the escalation of pain (through self-monitoring), fear avoidance, and the effect of pain catastrophising (cognitive-affective response to anticipated or actual pain) as well as managing self-perception of pain, thereby reducing reliance on pain medication. More research is clearly needed to explore some of the mechanisms behind how self-management techniques work in dealing with chronic conditions, as well as the people who are most likely to benefit.

### Implications

We also suggest that clinicians could consider the potential for an AT teaching service within some (but not all) pain clinics. Pain clinics that utilise a more medical model of pain are less likely to find a role for AT, but pain clinics working within a more psychosocial framework within a multi-disciplinary and multi-professional team might be more sympathetic to AT. An Alexander Technique service could add another dimension to the range of clinical, psychological and educational interventions that play a part in psychosocially-oriented pain clinics.

### Strengths and limitations

A strength of this study is its mixed methods design in combining quantitative and qualitative data to evaluate a healthcare service. Not only did we capture quantifiable changes in reported health status, medications and service utilisation, but through qualitative data we were also able to explain how those changes may have come about. This increased our confidence in putting forward explanations for the quantitative scores. Collecting either quantitative or qualitative data in isolation would have been less powerful. Moreover, the trend is often to publish each component separately, but as this paper shows the combination of datasets can lead to deeper understanding. We would recommend that future evaluations of health services include both qualitative and quantitative components and that these are reported together.

However, the perceived changes in outcomes cannot be attributed to the intervention due to the non-randomised, uncontrolled nature of the design. While the sample size for the study was relatively small and there was no control group to investigate whether changes would have occurred over time anyway, this before and after design is more common in service evaluations. In addition, service users were aware of the lessons they were receiving and this may have an impact on the reliability of self-reported results. However, a high quality RCT did report similarly positive results in health outcomes [23]. We did not carry out any sub-analyses on any groups within the study population, as the numbers of participants would be too small to construct reliable estimates and draw any meaningful conclusions. Further, the AT teachers administered the baseline and six week questionnaires, which could introduce another bias, although changes were sustained at three months, when the questionnaires were self-administered or administered by an independent researcher.

## Conclusions

Based on the results of this study we found that participants in AT lessons in a multi-disciplinary pain clinic reported modest improvements in health outcomes (pain severity and pain interference), and condition costs related to pain fell. Differences were found in how service users managed their pain, for example more than half stopped or reduced their medication. The qualitative data suggests that what had changed was the service users’ relationship to the pain and their pain management, and some individuals were more committed to maintaining their AT practice than others.

## Footnotes

1 StataCorp. 2009. *Stata Statistical Software: Release 11*. College Station, TX: StataCorp LP.
